# Retraction: Epidemiological insights into paratuberculosis in camels in Saudi Arabia: Bayesian estimation of true prevalence and identification of risk factors

**DOI:** 10.1371/journal.pone.0307546

**Published:** 2024-07-17

**Authors:** The PLOS ONE Editors

Following the publication of this article [1], concerns were raised about undisclosed competing interests between the corresponding author and the Editorial Board member who handled the manuscript through peer review [2–4].

In addition, during the editorial rereview of the article, PLOS noted that the ethics approval for this study was issued on the same day that the article was submitted to PLOS. PLOS does not consider that retrospective ethics approval is appropriate for animal research.

In light of the concerns above, which indicate this article breaches *PLOS ONE*’s Ethical Publishing Practice policies and *PLOS ONE*’s Animal Research policies, the *PLOS ONE* Editors retract this article. PLOS regrets that these issues were not identified prior to the publication of the article.

PK agreed with the retraction but stands by the article’s findings. AAN, MS, FH, KAMS, JH, MF, and AZ either did not respond directly or could not be reached.
